# Searching for Strangely Shaped Cookies – Is Taking a Bite Out of a Cookie Similar to Occluding Part of It?

**DOI:** 10.1177/0301006620983729

**Published:** 2020-12-30

**Authors:** Eli Brenner, Sergio Sánchez Hurtado, Elena Alvarez Arias, Jeroen B. J. Smeets, Roland W. Fleming

**Affiliations:** Vrije Universiteit Amsterdam, the Netherlands; Justus-Liebig-University Giessen, Germany

**Keywords:** object perception, shape perception, visual search, transformations, serial processing

## Abstract

Does recognizing the transformations that gave rise to an object’s retinal image contribute to early object recognition? It might, because finding a partially occluded object among similar objects that are not occluded is more difficult than finding an object that has the same retinal image shape without evident occlusion. If this is because the occlusion is recognized as such, we might see something similar for other transformations. We confirmed that it is difficult to find a cookie with a section missing when this was the result of occlusion. It is not more difficult to find a cookie from which a piece has been bitten off than to find one that was baked in a similar shape. On the contrary, the bite marks help detect the bitten cookie. Thus, biting off a part of a cookie has very different effects on visual search than occluding part of it. These findings do not support the idea that observers rapidly and automatically compensate for the ways in which objects’ shapes are transformed to give rise to the objects’ retinal images. They are easy to explain in terms of detecting characteristic features in the retinal image that such transformations may hide or create.

Objects in our environment are not always seen in their perfect form from the ideal viewpoint under uniform illumination. Objects’ retinal images are obviously influenced by transformations that affect the objects themselves, such as when a piece of paper is crumpled or torn or has text printed on it (Figure 1). However, objects’ retinal images are also influenced by transformations that do not affect the objects themselves, such as when a piece of paper is rotated with respect to the line of sight or when another object casts a shadow on it or occludes part of it. Despite all this, people are very good at identifying objects, somehow accounting for such transformations. How people deal with some such transformations has been studied quite extensively. For instance, there are many studies on how people manage to recognize objects by their colour (surface reflectance) despite the fact that changes in illumination transform the relative cone stimulations that underlie such judgments quite dramatically (colour constancy; Foster, 2011; Granzier et al., 2009). The ability to infer objects’ fundamental properties presumably somehow relies on identifying the basic prerequisites for an object’s identity and recognising whether other characteristics were caused by one of many possible transformations (Schmidt et al., 2016). This suggests that objects’ identities are inferred by combining some representation of their identity with the possible transformations that led to the current view (Spröte & Fleming, 2016). The challenging question is whether this is only true for transformations that change the view of the object, such as occluding, moving or rotating the object (Schmidt et al., 2016; Figure 1B), or also for transformations that change the object’s shape such as bending or crumpling them (Figure 1C).

**Figure 1.**
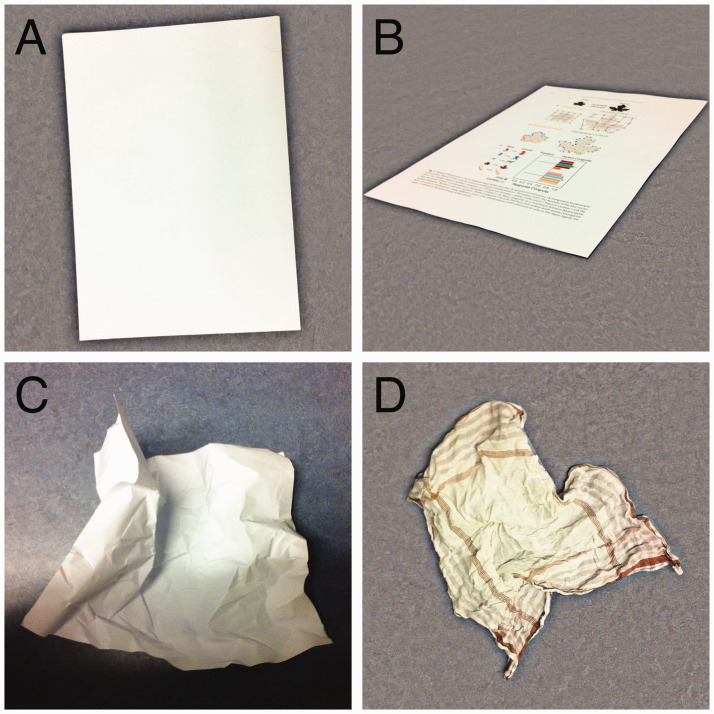
Objects and transformations. A: A piece of paper. B: The same piece of paper viewed at an angle, partly occluded by the text and images printed onto it. C: A similar piece of paper that is crumpled and unevenly illuminated. D: Not a piece of paper. We have little difficulty identifying that A–C are paper whereas D is not, but how?

People are quite good at identifying transformations that are likely to have been involved in converting one object into another (Schmidt et al., 2019) and can even classify unknown objects on the basis of the transformations that are likely to have been involved in making them (Fleming & Schmidt, 2019). When asked to indicate objects’ axes of symmetry, people are more inclined to treat segments that appear to be missing as parts of the object if the segments appear to have been “bitten” off, than if the segments were removed more smoothly (Spröte et al., 2016). Further support for transformations playing a role in judging shape is that when one shape is abruptly replaced by another, the change is more likely to be judged to have taken place gradually if the second shape could be created from the first by forcefully compressing a part of it than if it could not (Chen & Scholl, 2016). All these findings support the idea that objects’ identities can be separated from how they have been transformed, as illustrated in Figure 1.

It is unclear how early in the process of visual object recognition the separation between identity and transformation occurs. Leyton (1989, 1992) suggests that explicit inference of the “causal history” of objects is a central aspect of the way shape is represented, so that when viewing the transformed shape of an object, the visual system is able to access a representation of the object’s original shape. Chen and Scholl’s findings with apparent motion indicate that the visual system can rapidly and automatically infer transformations that unfold over time at least as early as the stage when apparent motion is computed. Yet it remains unclear whether inferences about the original form of transformed objects also occur rapidly and automatically, and whether separated representations early in visual processing are available or even dominate tasks such as visual search. That is the topic of the current study.

There is a long tradition of using visual search to measure how quickly various visual distinctions can be made (Treisman & Gelade, 1980; Wolfe, 1994, 2001). Here, we try to determine whether inferring what an object looked like before it was transformed occurs rapidly and automatically, early in the process of judging the object’s shape. We do so by measuring search times. One transformation that has been studied extensively using visual search tasks is occlusion. Studies on occlusion have shown that it is particularly difficult to find a simple shape with a section removed when the part that is missing coincides with the edges of another object, as if the part that is missing is hidden behind the other object (Rauschenberger & Yantis, 2001; Rensink & Enns, 1998). This can be interpreted in terms of the object being “completed” in early vision (Chen et al., 2018; Rensink & Enns, 1998). The completion of occluded parts of objects is compelling enough to fool people in magic tricks (Ekroll et al., 2018) and to make it easy to detect the orientation of a bar of which one can only see two corners (Figure 1c of Wolfe & Horowitz, 2004).

Several papers on the role of transformations in object recognition used a picture of a cookie from which a piece has clearly been bitten off as an example of an object that has been transformed without changing its identity (Chen & Scholl, 2016; Spröte et al., 2016; Spröte & Fleming, 2013, 2016). Biting off a piece of a cookie is somewhat similar to occluding part of it, because in both cases a part is removed from the visible image of the cookie. However, biting off a piece of the cookie changes the cookie’s actual shape, whereas occluding part of it only changes the view of the cookie. Moreover, when looking at an occluded cookie the cause of the transformation is visible: the occluder. When looking at a bitten cookie only the consequence of the transformation is visible: the missing part with bite marks. One reason for it being difficult to find the hidden part of the cookie might be that the border between the cookie and the occluder is assigned to the occluder rather than to the cookie (Spröte & Fleming, 2013). In this study, we examined to what extent cookies of which a part is occluded or has been bitten off are treated as if they were whole cookies during visual search. Are bitten cookies fundamentally different because when someone takes a bite out of a cookie the actual shape of the cookie changes rather than only the shape of its visible part? Or are bitten cookies treated as whole cookies because observers rapidly and automatically complete the missing parts, as they appear to do when a part of the cookie is occluded?

The study consisted of four sessions with three kinds of items in each session. The items in each session were whole cookies and two kinds of incomplete cookies. One of the two kinds of incomplete cookies appeared to be a transformed version of a whole cookie while the other did not. For each of the two transformations (occlusion or biting) either of the two kinds of incomplete cookie could be the target. For sessions in which the transformation was occlusion, we used whole cookies, cookies of which a part was occluded by a blue disk (*occluded*), and cookies of which the visible part had the same shape as the occluded ones, but the blue disk was some distance from the relevant edge (*separated*). If occluded cookies are completed in early vision, a *separated* target should be easy to find, irrespective of the number of whole and partially occluded cookies, because all other cookies are, or at least appear to be, whole. A partially *occluded* target cookie should be even more difficult to find than in previous studies, because neither the completed object nor the visible part is unique. For sessions in which part of the cookie could be bitten off, we used whole cookies, cookies missing a part because it was bitten off (*bitten*), and cookies that were baked in a shape that was similar to the shape of the cookie when part had been bitten off (*shaped*). Again, if the bitten part is completed in early vision the *shaped* target should be easy to find, irrespective of the number of whole cookies and ones missing a bite, whereas the *bitten* target should be difficult to find because neither the whole object nor the general shape of the visible part is unique.

## Methods

### Equipment and Images

**Figure 2.**
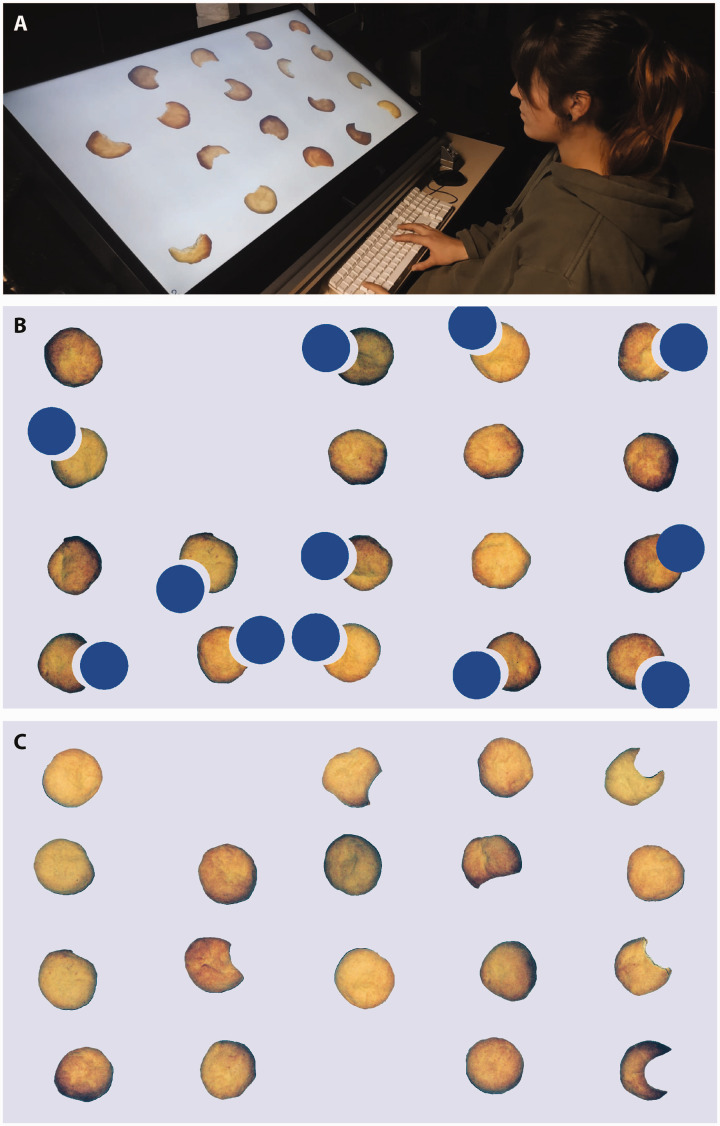
The setup and two examples of displays. A: The participant sat in front of the screen with his or her fingers on the “a” and “p” keys of a keyboard. B: Example of a display in which the target, a partially *occluded* cookie (third row, rightmost column), is presented together with 11 *separated* cookies and 6 *whole* cookies. C: Example of a display in which the target, a *bitten* cookie (at the same position as in B), is presented together with 5 *shaped* cookies and 12 *whole* cookies.

The stimuli were presented on a large screen (92 × 52 cm; 1,920 × 1,080 pixels). The screen was tilted towards the participant by 31° with respect to horizontal (Figure 2A). Each stimulus consisted of 18 images, jittered slightly with respect to a grid of 5 × 4 positions (leaving two randomly selected positions empty on each trial). The images were based on photographs of hand-made cookies on a uniform background. The cookies were photographed from above when lying on a flat surface, under diffuse illumination. We used 20 photographs of whole, more or less circular cookies, 20 of cookies baked with crescent-like shapes, and 21 of whole cookies from which a small portion had been bitten off. One advantage of using real cookies is that doing so ensures that the differences between the images include the natural cues that would be used to detect whether a section has been bitten off. Image processing was used to convert all the backgrounds to exactly the same grey as the background on the screen. Each photograph was cropped to the smallest square that would include the complete cookie. These squares were presented as 8.32 × 8.32 cm images at randomly selected positions within 1.53 cm of the centre of the appropriate grid position horizontally and 1.04 cm of the centre of the grid position vertically. Each image could be presented in any of the four possible orientations. The choice of photograph from within the relevant set as well as its position and orientation on the screen were determined at random for each image on each trial.

In the two sessions involving occlusion, we only used the photographs of whole cookies. There were three kinds of items: ones in which a cookie was partially occluded by a 6.24 cm diameter blue disk centred 3.9 cm from the centre of the photograph in a randomly chosen direction (*occluded*), ones in which a similar portion of the cookie was visible as when occluded but with the blue disk shifted from the concave edge by 1.04 cm (*separated*), and ones with no occluder (*whole*). When the target was *occluded*, the other items were either *separated* or *whole* (Figure 2B). When the target was *separated*, the other items were either *occluded* or *whole*. In the two remaining sessions (involving bitten cookies), we used all three kinds of photographs: the same photographs of whole cookies (*whole*), photographs of cookies from which a bite had been taken (*bitten*), and cookies that were baked in a more or less crescent shape (*shaped*). When the target was *bitten*, the other items were either *shaped* or *whole* (Figure 2C). When the target was *shaped*, the other items were either *bitten* or *whole*. Including whole cookies in all sessions provides participants with a reference for the untransformed cookies’ likely appearance (Chen et al., 2018).

There was always an even number of whole cookies (0, 2, 4, 6, 8, 10, 12, 14, or 16), so the number of incomplete cookies *n* was also even. When the target was present it was accompanied by n-1 other incomplete cookies (distractors: *separated* if the target was *occluded*; *occluded* if the target was *separated*; *shaped* if the target was *bitten*; *bitten* if the target was *shaped*) and by 18-*n* whole cookies. When the target was absent there were *n* distractors and 18-*n* whole cookies. The target and the distractors were always similar in terms of the shape of the visible part of the cookie and the presence or absence of a blue disk.

### Participants and Procedure

The study was conducted in accordance with the approval of the Ethics Committee of the Vrije Universiteit Amsterdam. Each of the 12 participants (all students; 7 women and 5 men) signed an informed consent form and then took part in 4 sessions: one for each of the four kinds of targets, in random order. Within each session, there were 8 practice trials, followed by 180 trials in which each of the 9 numbers of whole cookies was presented 10 times with the target present and 10 times with the target absent, all in random order. The participants’ task was to indicate whether a target was present or absent by pressing the “p” or “a” key of a keyboard that was lying between them and the screen (Figure 2A). Participants were encouraged to keep their fingers on the two keys and to answer as soon as they could.

### Analysis

We were primarily interested in the time it took participants to determine whether or not there was a target present in the display. We therefore measured the time between the moment the stimulus appeared and the moment the participant pressed one of the two keys. We then determined the median value of this time for each participant, kind of target, and number of incomplete cookies. We did so separately for trials in which the target was present and trials in which the target was absent, and plotted the means and standard errors of the 12 participants’ median values. We also determined how often participants pressed the wrong key, indicating that there was a target when there was none, or that there was no target when a target was presented.

To summarize the time it took participants to respond, we assumed that the times that we found could be described as some kind of serial search, and used the data to determine two times for each session. If *T_w_* is the time it takes to dismiss a whole cookie as a potential target, and *T_i_* is the time it takes to distinguish between the matching incomplete cookies (target and distractor), we can express the time taken to correctly identify that there is no target (for *n* incomplete cookies and some minimal time $t_{0}$) as:

(1) $$t_{\mathrm{absent}}=t_{0}+nT_{i}+(18-n)T_{w}$$

We also assumed that when the target is present, on average it would be found after having searched through half the nontarget items, such that

(2) $$t_{\mathrm{present}}=t_{0}+T_{i}+(\left(n-1\right)T_{i}+\left(18-n\right)T_{w})/2$$

To determine $T_{i}$ and $T_{w}$, we assume that $t_{0}$ is a constant, and fit Equations 1 and 2 to the data (linear least squares fit). We fit a single value of $t_{0}$ to all the data, with separate values of $T_{i}$ and $T_{w}$ for each session and participant. We then compared the values of $T_{w}$ for the paired sessions to see whether whole targets are more difficult to dismiss (higher values of $T_{w}$) when the occluded or bitten cookie was the target (as would be expected if these targets are seen as whole). We also compared the values of $T_{i}$ for the pairs of sessions in which the target and distractor were swapped (*occluded* versus *separated*; *bitten* versus *shaped*) to evaluate whether there were differences in $T_{i}$ depending on which of the same two items was the target. These comparisons were evaluated with paired *t* tests. To examine whether the values that we obtained in this manner provide a reasonable representation of the data, we accompanied the plotted search times with the lines showing how *t*_present_ and *t*_absent_ depend on *n* (the number of incomplete cookies) according to this model. For this we used the means of the 12 participants’ fit values of $T_{i}$ and $T_{w}$ for the condition in question.

## Results

**Figure 3.**
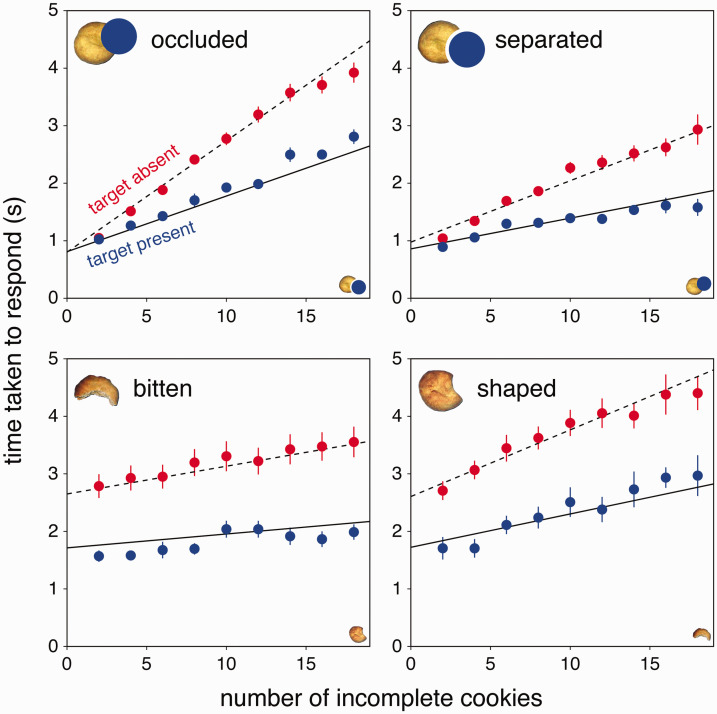
Search times when each kind of target was either present (blue symbols; solid lines) or absent (red symbols; dashed lines). The kind of target is indicated in the top left corner of each panel. A corresponding incomplete cookie is shown in the bottom right corner. The points with error bars are means and standard errors across individual participants median search times. The lines show *t_absent_* and *t_present_* from Equations 1 and 2 for the mean of the participants’ fit parameters.

We did not exclude any data. Although there were always 18 items on display, search times always increased with the number of incomplete cookies (Figure 3), showing that it was always easier to distinguish between the target and a whole cookie than between the target and a different incomplete cookie ($T_{i}>T_{w}$; the difference was smallest for the bitten cookies, leading to the shallowest slopes for that condition). The increase in search time with the number of incomplete cookies was more or less linear, in line with the serial search we assumed for our model. The equations based on this assumption (Equations 1 and 2) fit the data reasonably well (lines in Figure 3). We therefore feel justified in interpreting the fit values of $T_{i}$ and $T_{w}$, respectively, as the times taken to dismiss a whole cookie as a potential target and to determine whether or not an incomplete cookie is the target (Table 1). The fit value of $t_{0}$ is 613 ms.

**Table 1.** Estimate of the Time It Takes to Dismiss a Whole Cookie as a Potential Target ($T_{w}$) and to Distinguish Between the Two Kinds of Incomplete Cookies ($T_{i}$).

Target	$T_{w}$ (ms)	$T_{i}$ (ms)
Occluded	11 ± 7	204 ± 29
Separated	20 ± 7	127 ± 37
Bitten	113 ± 35	161 ± 46
Shaped	111 ± 29	227 ± 59

Means ± standard deviations across participants.

For *occluded* targets, the time taken to establish that a whole cookie was not the target (the value of $T_{w}$) was very short (11 ms). Consequently, response times were similar in the presence and absence of a target when there were few incomplete cookies and increased about twice as fast with the number of incomplete cookies when the target was absent than when it was present (Overvliet et al., 2007). The ease with which whole cookies were dismissed is inconsistent with the idea that occluded targets are perceived as whole. This is supported by the value of $T_{w}$ being slightly smaller when the target was *occluded* than when it was *separated* (mean difference of 9 ms; *t*_11_ = 5.01; *p* = .0004), rather than being larger as we had anticipated on the basis of occluded cookies being perceived as whole. However, whole cookies may have been easy to dismiss because they were not accompanied by conspicuous blue disks (see Figure 2B), and this may have been slightly more difficult for *separated* targets because the blue disk was slightly further away from its associated cookie. In the sessions with bitten cookies, it took about 100 ms longer to establish that whole cookies were not the target, with no systematic difference between *bitten* and *shaped* targets (mean difference of 2 ms; *t*_11_ = 0.50; *p* = .63).

**Figure 4.**
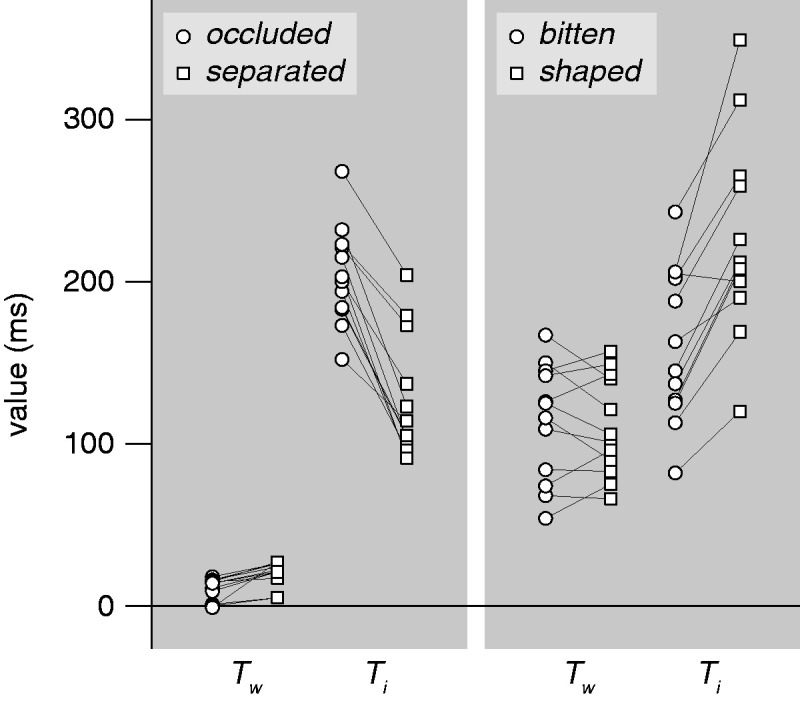
Individual values of *T_w_* and *T_i_*. Lines connect values for the same participant.

Whether the time taken to distinguish between target and distractor changed when their roles were swapped was also tested with paired *t* tests. It took considerably longer to make the distinction (larger values of $T_{i}$) when the target was a partially occluded cookie (*occluded* in Table 1 and Figure 4) than when there was a gap between the visible part of the cookie and the occluder (*separated*; mean difference of 77 ms; *t*_11_ = 9.97; *p* < .0001). In contrast, it took considerably shorter to make the distinction when the target was a cookie from which a small part had been bitten off (*bitten*) than when it was baked with a crescent-like shape (*shaped*; mean difference of 66 ms; *t*_11_ = 6.28; *p* < .0001).

The times that are shown in Figure 3 and that were used for the fits are means and standard errors of the medians of the ten replications per participant, irrespective of whether or not the response was correct (Table 2 in the Appendix shows the fit values when only trials with correct responses were considered). Participants seldom reported that there was a target when there was none: when the target was absent they indicated that it was present on fewer than 1% of trials when searching for an *occluded* or *separated* target, on 1.6% of trials when searching for a *bitten* target and on 3.9% of trials when searching for a *shaped* target. They did regularly miss the target when it was present: this happened on 5.1% of trials with a *separated* target, 8.5% of trials with an *occluded* target, 10.5% of trials with a *bitten* target, and 18.5% of trials with a *shaped* target. Not surprisingly, they missed more targets when there were more incomplete cookies (not shown). That they did regularly miss a target when it was present shows that participants tried to respond quickly, as instructed.

It is evident from the values of $T_{i}$ in Table 1 and Figure 4, that it took longer to distinguish between a partially occluded cookie (*occluded*) and a cookie of which a similar part was visible but with a shifted occluder (*separated*) when the *occluded* cookie was the target, but that it took less long to distinguish between a cookie with a part bitten off (*bitten*) and a cookie that was baked with a crescent shape (*shaped*) when the *bitten* cookie was the target. If bitten cookies were perceived as whole they should have been the most difficult to find, because there were both whole cookies and cookies with the same overall crescent-like shape amongst the other items, and there were no large blue disks to help dismiss whole cookies. The finding that it takes less time to distinguish between bitten and shaped cookies (smaller value of *T_i_*) when the bitten cookie is the target is consistent with bitten cookies having some feature that the shaped ones lack (Wolfe, 2001). Presumably, the ragged edge with a different local contrast caused by the bite is such a feature.

## Discussion

To evaluate whether considering the transformations that gave rise to an object’s retinal image is fundamental to quickly identifying the object, we examined to what extent taking a bite out of a cookie is similar to occluding part of it in terms of the time needed to find the cookie. Both transformations remove part of the cookie from sight, and neither transformation changes the identity of the cookie in a fundamental manner. As expected, it took more time to find an occluded cookie amongst separated ones, than to find a separated cookie amongst occluded ones (*T_i_* was larger for *occluded* than for *separated* targets; data in top panels of Figure 3). We expected this because we expected the occluded cookie to be treated as whole, and finding a single whole cookie amongst ones missing a part is more difficult than finding the one cookie with a part missing (Wolfe, 2001). We had expected the occluded cookie to be particularly difficult to find in our study, because there were also whole cookies that were not occluded, so looking for a whole cookie is not enough. Contrary to the idea that taking a bite out of a cookie is similar to occluding part of it, it did not take more time to find a cookie with a piece bitten out of it than one that was baked in a similar shape. The bite marks even appeared to help participants quickly detect the bitten cookie (*T_i_* was smaller for *bitten* than for *shaped* targets; bottom panels in Figure 3).

Most of our findings for occlusion are consistent with those of the previous studies that inspired our approach. There are many circumstances in which partially occluded shapes appear to be treated as if they were completed in visual search (Rauschenberger & Yantis, 2001; Rensink & Enns, 1998), although such completion does not make the shape fully equivalent to the shape without occlusion (Wolfe et al., 2011). Only one of our findings was inconsistent with previous results. Rensink and Enns (1998) found that it took much longer to determine that a whole item was not the target when the missing part that defined the target looked as if it was occluded than if it did not. We did not confirm this: *T_w_* was even smaller for an *occluded* than for a *separated* target. This inconsistency could be because different features were used to dismiss items in their study than in ours. An obvious possibility is that our whole cookies were easy to dismiss because there was no blue disk near them. This is probably not the whole answer, because Rensink and Enns (1998) report a value of 8 ms per item for dismissing whole items when looking for *separated* targets, which is even less than our value of 20 ms for *T_w_*. In their study, all the whole items were accompanied by similar objects to the occluder. Maybe the fact that our items were not all accompanied by a potential occluder made participants consider them differently. One reason to suspect that the presence of an occluder was used for a quick screening in our study is that it appeared to be more difficult to dismiss whole cookies when the occluder was further away (*T_w_* is larger for a *separated* target than for an *occluded* one).

We already knew that occluded items are not treated precisely as whole ones, even if there is an object like the occluder near the whole targets. Rensink and Enns (1998) report that distinguishing a whole item from a separated one is faster than distinguishing an occluded one from a separated one (as we also find: *T_w_* was much smaller than *T_i_* for our *separated* targets). Similarly, Rauschenberger et al. (2004) found that it was easier to distinguish a *separated* item from a whole one than from a partially occluded one. Rauschenberger and Yantis (2001) proposed that it takes some time for the untransformed representation to override other representations (filling in the occluded parts through amodal completion). Thus, although much of the evidence is consistent with occluded items being treated as whole, it is evident that an occluded item is not completely equivalent to the corresponding item without occlusion. This is not a problem for the interpretation of our results, because our interpretation relies on the asymmetry in search times that arises from completing the item rather than on a complete equivalence.

The fact that the *occluded* cookie was so easy to distinguish from whole ones (see value of *T_w_* for *occluded* targets in Table 1) might be due to the absence of the blue occluder, but the asymmetry in distinguishing between *occluded* and *separated* targets, depending on which is the target (see values of *T_i_* for these targets in Table 1) cannot be. In accordance with earlier studies, we interpreted this asymmetry as indicating that occluded cookies are considered in their untransformed state to some extent. However, this interpretation rests on the assumption that the asymmetry arises from some kind of completion. An alternative explanation is that the gap between the item and the occluder in the *separated* items is itself a feature that can be detected.

There are two ways in which considering transformations could be important for object perception. The first considers the sequence of transformations that were involved in making the object (Leyton, 1989). The processes involved in making an object are at least partially responsible for its shape, so transformations that leave a mark on the object can be expected to contribute to the object’s identity. It has been demonstrated that people can identify objects that have undergone similar transformations (Fleming & Schmidt, 2019; Schmidt & Fleming, 2018). However, this does not necessarily mean that objects can be identified by the transformations that were involved in creating them, because different objects can be created using similar transformations. This study only considers a second way in which considering transformations could be important for object perception: by compensating for how transformations might have influenced the visible shape of objects that have already been made. There may be a difference between transformations that only change what you see, such as occluding part of an object or rotating it in depth, and transformations that change the object itself, such as crumpling a piece of paper or biting off a piece of a cookie. If the initial percept is sensitive to this distinction, we might judge the *occluded* cookie to be whole, but the *bitten* cookie no longer to be whole. In that case, the *bitten* cookie may be easier to find because it is the only incomplete cookie. The *shaped* cookies that resemble the *bitten* ones in overall shape are more difficult to dismiss as targets than are the *whole* ones (Figure 4 and Table 1) because of their shape, but the fundamental distinction is that the *bitten* cookies are incomplete. However, if this were the main distinction we would expect the *shaped* cookies to be found faster the more *bitten* targets were present, because the bitten targets would be easy to dismiss. That was not the case (Figure 3).

Thus, visual search does not always make use of a mechanism that infers what an object looked like before it was transformed rapidly and automatically, early in the process of judging the object’s shape. Actually, it is easy to explain our findings within the feature-based view of visual search. In the case of occluded objects being more difficult to find, one could consider that it is the gap itself that is easy to find (Rosenholtz, 1999). In Figure 2B, this gap seems to stand out. The slope for detecting a cookie separated from a potential occluder by a gap is not zero, but the presence of the gap is presumably easier to find than the absence of such a gap (Rosenholtz, 2001; Wolfe, 2001), just as a concavity is easier to find than the absence of a concavity (Hulleman et al., 2000). Similarly, both the corrugations (made by the teeth) and the lack of change of colour (that normally occurs at the edges of the cookies during the baking process) where the part has been bitten off a cookie are probably easier to find than the absence of such features. Consequently, the distinction between the *bitten* and *shaped* cookies is both faster and accompanied by fewer errors when the *bitten* cookie is the target (Figure 4).

The fact that there are low-level features that could identify the target does not mean that our findings are trivial, because if search in our tasks had relied on shapes that had been completed at an earlier stage, such features might not be available for performing the task (as suggested by the interpretation of many of the cited studies involving occlusion). There are cases in which features are lost to visual search. Surface slant can be judged from various features, including gradients in binocular disparity and how retinal image shapes differ from object shape through perspective, but search times depend on the extent to which the target differs from other items in the slant that is judged by combining such features, rather than on the extent to which the features themselves differ (Sousa et al., 2009). If objects’ original shapes had been determined in a manner that considered the transformations used in this study before the search began, a similar search asymmetry should have been found for the bitten cookie as for the occluded one. Our results are more consistent with search relying on features such as the gap and the bite marks, in which case the opposite asymmetries are easy to explain because the occluded cookies miss the gap, whereas the bitten ones have bite marks.

## Conclusions

Looking for a cookie of which a part has been bitten off is very different from looking for one of which a part is occluded. Observers certainly do not treat bitten cookies as whole cookies. They probably do not treat partially occluded cookies as whole cookies either.

## Appendix

The same overall pattern of results as that seen in Table 1 is also observed if only correct trials are considered.

**Table 2.** Values Determined as for Table 1, but Excluding Trials in Which the Participant Responded Incorrectly.

Target	$T_{w}$ (ms)	$T_{i}$ (ms)
Occluded	30 ± 6	217 ± 30
Separated	37 ± 7	143 ± 37
Bitten	130 ± 35	173 ± 44
Shaped	128 ± 32	232 ± 56

The fit value of $t_{0}$ is 353 ms.
